# Influence of environmental awareness on the willingness to pay for green products: an analysis under the application of the theory of planned behavior in the Peruvian market

**DOI:** 10.3389/fpsyg.2023.1282383

**Published:** 2024-01-05

**Authors:** Elizabeth Emperatriz García-Salirrosas, Manuel Escobar-Farfán, Ledy Gómez-Bayona, Gustavo Moreno-López, Alejandro Valencia-Arias, Rodrigo Gallardo-Canales

**Affiliations:** ^1^Faculty of Management Sciences, Universidad Autónoma del Perú, Lima, Peru; ^2^Department of Administration, Faculty of Administration and Economics, University of Santiago of Chile, Santiago, Chile; ^3^Faculty of Business, Universidad de San Buenaventura, Medellín, Colombia; ^4^Faculty of Business, Institución Universitaria Marco Fidel Suarez, Bello, Colombia; ^5^Escuela de Ingeniería Industrial, Universidad Señor de Sipán, Chiclayo, Peru; ^6^Departamento de Tecnologías de Gestión, Facultad Tecnológica, Universidad de Santiago de Chile, Santiago, Chile

**Keywords:** willingness to pay, green products, eco-friendly products, theory of planned behavior, environmental awareness

## Abstract

**Introduction:**

This paper aimed to build a predictive model through an empirical study to examine the influence of environmental awareness (EA) on attitude (ATT) and perceived behavioral control (PBC), as well as to determine the influence of the three variables of the theory of planned behavior (TPB) on willingness to pay (WP) for green products in the Peruvian market.

**Methods:**

A total of 405 Peruvian consumers were surveyed. Most of them were between the ages of 18 and 30 and single. To test the hypotheses, partial least squares (PLS-SEM) were used using the SamrtPls4 software. The results show the significant positive effect of EA on ATT and PBC. The positive and significant effect of ATT, SN, and PBC on WP was also tested A total of 405 Peruvian consumers were surveyed. Most of them were between 18 and 30 years old and single. To test the hypotheses, partial least squares (PLS-SEM) was used using SamrtPls4 software.

**Results:**

The results show the positive and significant effect of AD on ATT and PBC. The positive and significant effect of ATT, SN and PBC on WP was also tested.

**Discussion:**

The research provides antecedents that allow evaluation of the possibility that companies and governments adjust the dissemination strategies and related public policies regarding the impact of environmentally responsible behavior in order to contribute to the development of environmental awareness as a variable that promotes the disposition of consumers to pay for environmentally friendly products.

## 1 Introduction

The growing relevance of environmental concerns in modern society has had a noticeable effect on consumer behavior (Horani and Dong, 2023). Latin American countries have seen an increasing preference towards sustainable consumption and higher awareness of the environment (Severo et al., 2021; Valenzuela-Fernández et al., 2022; García-Salirrosas et al., 2023; Gómez-Bayona et al., 2023; Valenzuela-Fernández et al., 2023). Therefore, it is essential to understand how environmental awareness influences purchasing decisions (Hlaváček et al., 2023). Consumer behavior can considerably affect the demand for sustainable products, which contributes to reducing the environmental impact associated with production and consumption (Bósquez et al., 2022). In this scenario, it is crucial to consider that consumer behavior influences the demand for sustainable products, which helps reduce the environmental impact of production and consumption. As a result, companies and policymakers must understand how this awareness of sustainability affects consumer purchasing behavior (Xu et al., 2020; Ali et al., 2021; González-Viralta et al., 2023).

Some countries, including Peru, have regulations that promote sustainability and environmental responsibility (Adaui and Cristian, 2020). In this scenario, companies that adopt sustainable practices can gain competitive advantages by meeting the demand of increasingly environmentally conscious consumers (Kumar et al., 2021; Severo et al., 2021). This knowledge can help them to offer products that align with the values and preferences of their target customers and ultimately enhance their profitability (García-Salirrosas and Rondon-Eusebio, 2022; Gómez-Bayona et al., 2023). In this way, environmental awareness is crucial in shaping consumer preferences toward sustainable products. Awareness refers to understanding the connections between our daily actions and the environment (Xu et al., 2019). This research aims to investigate the impact of environmental awareness on consumers’ willingness to pay for green products in a culturally rich and biodiverse country. Studying this topic in the Peruvian context is especially relevant since each country may have particularities regarding environmental awareness, consumer culture, and socioeconomic factors that affect purchasing decisions.

Since the beginning of the COVID-19 pandemic in October 2019, some environmental concerns have been generated for society (Ekawati et al., 2023). One of those that stands out is the awareness of consumption aspects of organic and green products that benefit health and the environment (Hoejmose and Adrien-Kirby, 2012; Popescu, 2020; Bou and Sánchez, 2023). In the same way, it has arisen with the use and preference of green products or services that positively impact the environment (Ekawati et al., 2023). This change in the paradigm has motivated the planning and execution of strategies during and after the COVID-19 pandemic from economic, social, environmental, and cultural points of view (Da’ar and Kalmey, 2023). There is increasing evidence of a growing preference for sustainable consumption and greater environmental awareness in Latin American countries (Severo et al., 2021; Valenzuela-Fernández et al., 2023). Therefore, studying this topic in the Peruvian context is particularly relevant, as each country may have unique characteristics regarding environmental awareness, consumer culture, and socioeconomic factors that influence purchasing decisions.

The intention of the younger generations to purchase green and healthful products has become more apparent. Decades ago, there was no concern regarding this issue; however, care for the environment is being pursued related to the limitation and quantity of natural resources (Pérez and Hernández, 2020; Alvarez-Risco et al., 2021; Kumar et al., 2021; Valenzuela-Fernández et al., 2022; Bósquez et al., 2023). In this context, consumer purchasing behavior is influenced by economic, political, ahoyosdnd cultural factors and environmental factors (Bertram and Chi, 2017). However, society has yet to reach a consensus on the most important factor, as some consumers may prefer products with more ecological manufacturing processes (Müller-Pérez et al., 2022). In contrast, others may choose the final product without regard to the manufacturing process (Fuentes, 2015). Therefore, society and organizations struggle to identify the factors that influence the selection of an eco-friendly product. Society and organizations are currently focused on identifying the factors that impact the selection of eco-friendly products (Lee, 2011; Kumar and Ghodeswar, 2015). This research aims to answer the question of whether environmental awareness affects the willingness of consumers to pay for green products in the Peruvian market.

The present study applies Ajzen’s (2002) theory of planned behavior to understand the phenomenon of sustainable product choice better. This conceptual framework examines attitudes, subjective norms, and how people think they can control their behavior. It gives us a solid foundation for why people choose sustainable products. This study applies the theory of planned behavior as a conceptual framework because we need to do more than describe the relationship between environmental awareness and willingness to pay. We need to understand what drives people to act in these ways. This theoretical approach is widely recognized in consumer psychology, providing a solid framework for unraveling the complexities of attitudes, subjective norms, and perceptions of control that influence sustainable purchasing decisions (Xu et al., 2020; Valenzuela-Fernández et al., 2023). Therefore, the main focus of this study is to investigate how environmental awareness (EA) influences attitude (ATT) and perceived behavioral control (PBC), and how the three variables of the theory of planned behavior (TPB) impact the willingness to pay (WP) for green products in the Peruvian market.

The article’s structure follows: The introductory section overviews the research context. The second section conducts a thorough literature review, examining theories, studies, and prior approaches relevant to our research topic. The third section outlines the methodology employed, detailing the techniques and procedures for data collection. The fourth section then presents the outcomes of the thorough analysis with supporting empirical evidence. The fifth section discusses these results, contextualizing the findings of the reviewed literature. Finally, the sixth section concludes the article by summarizing key findings, addressing limitations, and suggesting potential avenues for future research. This structured approach aims to provide a comprehensive and coherent understanding of the conducted research.

## 2 Literature review

### 2.1 Research variables

#### 2.1.1 Willingness to pay for green products

Willingness to pay is conventionally conceptualized as the highest price a consumer desires for a product or service (Wertenbroch and Skiera, 2002; Ioana-Daniela et al., 2018; Schmidt and Bijmolt, 2020). Studying consumers’ Willingness to Pay is critical for a better understanding of business and academic areas of their impact on learning purchase intention (Schmidt and Bijmolt, 2020; Becerril-Castrillejo and Muñoz-Gallego, 2022a; Becerril-Castrillejo and Muñoz-Gallego, 2022b). Willingness to pay is based on the individual’s value factor, particularly regarding sustainable products and industries. Therefore, costs or prices are drivers of motivation to pay (Yadav and Pathak, 2017; Walcher and Ihl, 2020).

Recent studies have affirmed that environmental awareness and sustainable consumption have increased among the population during the COVID-19 pandemic (Ali et al., 2021; Severo et al., 2021; Valenzuela-Fernández et al., 2022; Valenzuela-Fernández and Escobar-Farfán, 2022; García-Salirrosas et al., 2023; Gómez-Bayona et al., 2023; Valenzuela-Fernández et al., 2023). Therefore, it could suggest that consumer willingness to pay for ecological products has been strengthened or increased. COVID-19 has modified individuals’ purchasing of healthier and quality products (Wang et al., 2020). Also, quarantines and confinements have increased the desire to pay for green spaces to help the environment (Manso et al., 2021). For instance, González-Viralta et al. (2023) have affirmed that adopting environmentally friendly practices in supermarkets within the context of Chilean economics has a direct and favorable impact on customer willingness to pay for green products. Also, Prakash et al. (2019) and Kumar et al. (2021) highlighted that young environmentally conscious consumers are quickly paying a high price for green packaging. People generally intend to pay for a product or service to be more environmentally friendly (Doli et al., 2021). In this context, green consumption has become increasingly significant within the business sector and the academic community (Bósquez et al., 2023). Hence, a positive relationship was found between behavioral intention and willingness to pay.

#### 2.1.2 Environmental awareness

Environmental awareness encompasses understanding ecological issues and the fundamental relationships contributing to environmental impact (Milfont and Duckitt, 2004; Valenzuela-Fernández et al., 2022; García-Salirrosas et al., 2023; Guo and Xiao, 2023). This perspective emphasizes the importance of individual perception and knowledge concerning environmental problems, influencing subsequent behavioral choices (Xu et al., 2020; García-Salirrosas et al., 2023). As a result, environmental awareness predicts pro-environmental behavior (Kaiser et al., 1999; Omarova and Jo, 2022). It is consequently regarded as a crucial factor influencing human consumption patterns, actions, sustainability behavior, and environmental stewardship (Kaiser et al., 1999; Ali et al., 2021; Severo et al., 2021; Si et al., 2022). According to Bósquez et al. (2022), the strategic development of marketing campaigns is crucial in raising consumer awareness regarding the environmental consequences of consuming traditional products.

Previous studies have emphasized the importance of environmental awareness (Dharmaraj et al., 2021; Garg, 2021; Severo et al., 2021; Ferreira et al., 2023; García-Salirrosas et al., 2023; Valenzuela-Fernández et al., 2023). Psychological elements, such as attitudes and environmental awareness, significantly promote green consumption (Sun et al., 2019). For instance, Garg (2021) demonstrated that environmental knowledge significantly and positively influences intentions to make green purchases. On the other hand, García-Salirrosas et al. (2023) have asserted that there is an increased environmental awareness in consumption and a heightened intention to purchase environmentally friendly products in Latin American countries since the COVID-19 pandemic. Similarly, Dharmaraj et al. (2021) indicate that environmental awareness is a crucial variable in green marketing, given its direct impact on ecological purchase decisions. Furthermore, Ferreira et al. (2023) affirm that environmental awareness positively impacts the intention to recommend and behavioral intention not only in products but also in using technologies that contribute to environmental conservation.

#### 2.1.3 Theory of planned behavior

Over recent decades, the theory of planned behavior (TPB) has emerged as a highly dependable and precise framework for predicting and examining individual environmental behavior (Liobikienė et al., 2016; Verma and Chandra, 2018; Gholamrezai et al., 2021; Kumar et al., 2021; Farrukh et al., 2023; Savari and Khaleghi, 2023). According to Ajzen (1991, 2020), an individual’s behavior is determined by attitude, subjective norms, and perceived behavioral control. Attitude (ATT) is a theoretical framework that explains the positive or negative evaluations of cognitive beliefs about notions, persons, occurrences, or actions (Ajzen, 2002, 2015). Therefore, it may be inferred that when individuals exhibit a more favorable attitude towards specific conduct, their intention to engage in that behavior is more likely to grow (Armitage and Conner, 2001; Ajzen, 2002; Ajzen, 2020). Subjective norms (SN) communicate the social pressure on the individual to do something (Ajzen, 1991, 2020). According to Chiu et al. (2018), close relationships should influence an individual’s behavior, such as family, friends, neighbors, or co-workers (Ajzen, 1991, 2020; Madden et al., 1992). Lastly, perceived behavioral control (PCB) is defined as a predictor that reflects the ease or difficulty of the individual’s perception to perform a particular behavior (Ajzen, 1991; Madden et al., 1992).

### 2.2 Conceptual model and research hypothesis

**Figure 1:**
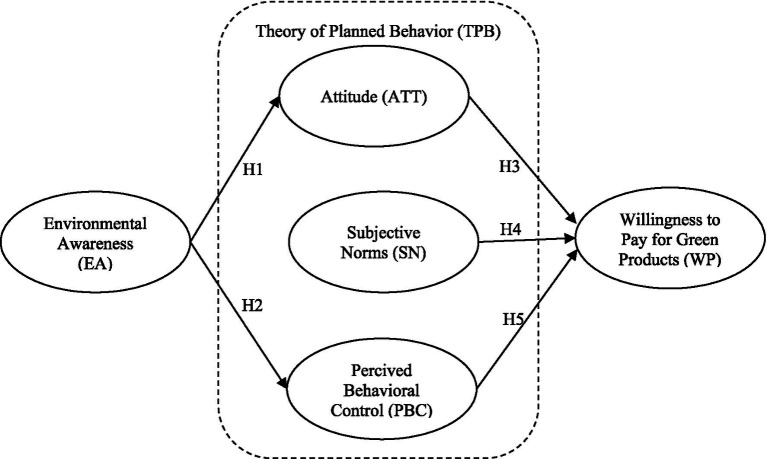
Conceptual model.

The theoretical background of this study is based on the formative model (Diamantopoulos and Winklhofer, 2001). Within the specific context of this research, we have formulated six hypotheses that center on consumers’ willingness to pay for green products. This proposed model provides a comprehensive framework that guides our exploration of environmental awareness influencing consumer behaviors, particularly in green product preferences. Figure 1 visually represents the relationships posited by our hypothesis, enhancing our research framework’s clarity and communicative power.

#### 2.2.1 Influence of environmental awareness on the theory of planned behavior

Previous studies have reported that awareness of the environment influences pro-environmental behavior (Xu et al., 2020; Valenzuela-Fernández et al., 2023). The first dimension of the theory of planned behavior is the attitude; there is evidence that indicates a positive relationship between environmental awareness and attitudes toward environmental behavior and the likelihood of individuals engaging in green purchasing (Arundati et al., 2020; Valenzuela-Fernández et al., 2022; García-Salirrosas et al., 2023; Gómez-Bayona et al., 2023; Hlaváček et al., 2023). For instance, Arundati et al. (2020) point out that individuals with a more environmentally solid awareness are more likely to acquire goods with a reduced environmental impact. Also, Wierzbiński et al. (2021) argue that as knowledge of environmental problems increases, the likelihood of adopting ecological practices, such as a preference for purchasing organic products, increases. This perspective implies that environmental awareness is a precursor to more sustainable consumption and purchasing decisions. Additionally, the study conducted by Valenzuela-Fernández et al. (2022) has recently determined that the COVID-19 pandemic has a significant role in shaping individuals’ intentions and attitudes to engage in environmentally responsible behavior. There is a pressing requirement for developing items manufactured using ecologically conscious techniques, enabling enhanced opportunities for recycling and promoting sustainable practices. Also, Hlaváček et al. (2023) have stated that awareness and attitudes encompass factors such as consciousness and concern for the environment and climate change, satisfaction with the present condition, and sufficient knowledge about environmental preservation. Based on this background, we propose the following hypothesis:

*H1*: Environmental awareness (EA) positively influences attitudes toward the consumption of green products.

Previous literature provides evidence to support the hypothesis that environmental awareness influences perceived behavioral control in the context of consumer behavior toward green products (Nekmahmud and Fekete-Farkas, 2020; Xu et al., 2020; Nekmahmud et al., 2022; García-Salirrosas et al., 2023; Valenzuela-Fernández et al., 2023). Xu et al. (2020) suggest a favorable relationship between environmental awareness and perceived behavioral control. Their study’s findings support the extensive application of the theory of planned behavior in examining consumer intentions and behaviors. Likewise, Tu and Yang (2019) emphasize that environmental conscientiousness and adopting technology products influence customers’ behavioral preferences. This means that people with greater environmental awareness are inclined to incorporate environmentally friendly items into their consumption options, establishing a connection with perceived behavioral control. Ahmed et al. (2021) also found a positive relationship between perceived behavioral control and intention to purchase organic food. Environmental awareness in this context reinforces the idea that environmental knowledge and sensitivity can increase consumers’ perception of control over their purchase decisions for sustainable products. In the same context, Valenzuela-Fernández et al. (2022) propose that the COVID-19 pandemic improved environmental awareness in nations such as Chile, Colombia, Peru, and Mexico. Therefore, in this case, ecological awareness supports the idea that attitude and knowing about the environment may give customers more power when choosing environmentally friendly products. Drawing upon the previously discussed background information, we propose the subsequent hypothesis:

*H2*: Environmental awareness (EA) positively influences perceived behavioral control (PBC) for the consumption of green products.

#### 2.2.2 Influence of the theory of planned behavior on willingness to pay for green products

Previous research has demonstrated that people who have a favorable attitude toward pro-environmental issues are more likely to engage in activities and actions that are beneficial to the environment (Zsóka et al., 2013; Davison et al., 2014; Bazrbachi et al., 2017; Sánchez et al., 2018; Valenzuela-Fernández et al., 2023). Ong et al. (2021) have suggested that individuals are inclined to favor and purchase environmentally friendly products in response to the COVID-19 pandemic. Kumar et al. (2021) and Valenzuela-Fernández et al. (2023) have indicated that since the beginning of the pandemic, there has been a noticeable rise in the inclination to purchase sustainable items. The potential for an enhanced inclination toward acquiring environmentally friendly items has been suggested as a factor that might contribute to an increase in the adoption of such products (Canova et al., 2020; Chen et al., 2022; Wang and Li, 2022). For example, university-age millennial consumers have very positive attitudes toward the consumption of green products when their environment significantly impacts their purchasing decisions (Bósquez et al., 2022, 2023; Hoyos-Vallejo et al., 2023). In this context, if a person has a favorable attitude toward green products, he is more likely to be willing to pay for them. Based on the above talks, the following hypothesis is put forth:

*H3*: Attitude (ATT) positively determined the consumers’ willingness to pay for green products (WP).

Secondly, subjective norms communicate the social influence pushed on an individual to perform behaviors (Ajzen, 1991; Chiu et al., 2018; Ajzen, 2020). In this sense, family, relatives, or friends should affect an individual’s behaviors (Ajzen, 2002; Rehman et al., 2019; Alexa et al., 2021). In the context of consumer behavior, it is common for individuals to consider the expectations and suggestions of their social network when making environmental purchasing decisions (Xu et al., 2020; Vu et al., 2021; García-Salirrosas et al., 2023). Narwal and Nayak (2019) have pointed out that social pressure affects willingness to pay. For instance, Yadav and Pathak (2017) have stated that Subjective norm positively influences the consumer’s intention to buy green products. Upon the emergence of COVID-19, some previous studies have investigated the impact on subjective norms increasing the intention of environmentally responsible behavior (Hidayat et al., 2021; Ong et al., 2021; Vu et al., 2021; Zebardast and Radaei, 2022). The pandemic has allowed people to learn about caring for the environment, and their environmental awareness has increased, resulting in social pressure for pro-environmental purchase behavior (Zebardast and Radaei, 2022). Hence, it is postulated that individuals who perceive a positive evaluation of green products within their social group are more inclined to demonstrate a willingness to pay costs for their acquisition. Based on the preceding discourse, the following hypothesis is posited:

*H4*: Subjective norms (SN) determined the consumers’ willingness to pay for green products (WP).

Thirdly, perceived behavioral control is a predictor encompassing the ease or difficulty of engaging in a specific action (Ajzen, 2020; 2002). Previous research indicates that perceived behavioral control positively influences the purchase intention and willingness to pay for eco-friendly (Lee and Back, 2007; López-Mosquera, 2016; Sánchez et al., 2018; Rehman et al., 2019; Lucarelli et al., 2020; Xu et al., 2020; Ngah et al., 2021; Vu et al., 2021). According to Sun et al. (2022), perceived control can be defined as the level of control an individual has over a particular behavior. Hence, in the context of consumption and purchase of ecological products, the greater the individual’s time, resources, and opportunities, the greater their perception of control over external factors and their propensity to purchase green products. Research conducted during the period the COVID-19 pandemic has indicated a significant correlation between perceived behavioral control and the inclination to purchase sustainable and green products (Lucarelli et al., 2020; Alexa et al., 2021; Zebardast and Radaei, 2022; Valenzuela-Fernández et al., 2023). An increasing number of individuals are concerned about the preference for eco-friendly products and climate change, raising their awareness of these issues during periods of pandemics (Lucarelli et al., 2020; Severo et al., 2021; Asif et al., 2022; Valenzuela-Fernández et al., 2022; Lavuri et al., 2023). Therefore, individuals who perceive that they have control over their purchasing decisions about green products are more willing to pay for such products. Based on the previous review, this study proposes the following hypothesis:

*H5*: Perceived Behaviour Control (PBC) determined the consumers’ willingness to pay for green products (WP).

## 3 Methods

### 3.1 Contex y method

This article aimed to build a predictive model through an empirical study to examine the influence of environmental awareness (EA) on attitude (ATT), subjective norm (SN), and perceived behavioral control (PBC), as well as determine the influence of the three variables of the theory of planned behavior (TPB) on the willingness to pay (WP) for green products in the Peruvian market. The study was conducted under a quantitative approach, non-experimental, and cross-sectional design, for which a self-administered questionnaire was applied (Hair et al., 2019).

### 3.2 Sample y procedure

For the data collection of this research, a non-probabilistic convenience sampling was applied (Hair et al., 2013). For this purpose, an online survey was conducted through the Google form, the link to which was shared using the WhatsApp application. The survey was applied during the period from June 29 to September 10, 2021, in Lima city, Peru. The research was focused on consumers from 18 years of age and could be male or female. It was only necessary that each person was willing to participate, that is why in order for the respondents to participate in the survey, they were informed that their participation was voluntary, that the data collected would be analyzed anonymously and that they would be used exclusively for academic and research purposes. About 700 Peruvian consumers were invited to participate in this survey; however, only 405 fully completed questionnaires were obtained, and these were deemed legitimate for the purposes of conducting this document’s statistical analyses. Of these, the largest number of participants were between 18 and 30 years old (46.2%) and their marital status were single (64.9%), and they had family income up to 2 minimum monthly wages (43.7%) (see Table 1).

**Table 1:** Sociodemographic data of the sample (*n* = 405).

	Categories	Frequency	%
Gender	Male	162	40.0
	Female	240	59.3
	I prefer not to say	3	0.7
Age range	18–30 years	187	46.2
	31–45 years	125	30.9
	46–55 years	58	14.3
	56 and over	35	8.6
Civil status	Married	99	24.4
	Cohabitant/Free union	26	6.4
	Divorced	13	3.2
	Single	263	64.9
	Widower	4	1.0
Academic level	High School/School	115	28.4
	Doctorate/PhD	12	3.0
	Specialization	50	12.3
	Master’s degree	42	10.4
	Undergraduate	160	39.5
	Preparatory	26	6.4
Family income	Up to 2 minimum wages	177	43.7
	From 3 to 4 minimum wages	129	31.9
	From 5 to 10 minimum wages	71	17.5
	From 11 to 20 minimum wages	19	4.7
	Greater than 20 minimum wages	9	2.2

### 3.3 Measurements

The construct created by Kumar et al. (2021) was employed in the development of this study model. The questionnaire was composed of a total of 18 items, distributed to assess attitude (3 items), social norm (3 items); perceived behavior control (3 items) and willingness to pay (3 items). and to evaluate the environmental awareness variable, the construct developed by Severo et al. (2021) was used (6 items). All items are assessed using a Likert-type scale, ranging from 1 to 5 points, where 1 means “Strongly Disagree” and 5 means “Strongly Agree.” The digital questionnaire was divided into two parts. The first section presented the 18 items already mentioned, and the second section was made up of questions to collect sociodemographic data of the participants, such as age, sex, marital status, and others.

### 3.4 Data analysis

To perform the statistical analysis of the data, Partial Least Square PLS-SEM was used to test the hypotheses. PLS-SEM is a comprehensive multivariate statistical analysis approach that includes measurement and structural components to simultaneously examine the relationships between each of the variables in a conceptual model, which has the characteristic of multivariate analysis, that is, it involves a number of variables ≥3 (Hair et al., 2013). In addition, PLS-SEM was employed in the present study because it facilitates theory building (Hair et al., 2011). SmartPls (Version 4.0) was used to perform the PLS-SEM analysis.

## 4 Results

In order to evaluate with the PLS-SEM, we took two stages: (1) Evaluation of the measurement model and, (2) Evaluation of the structural model. The first moment involves assessing the validity and reliability of the measurement model, this step evaluates the relationships between each construct with its associated items and, the second moment evaluates the structural model, which deals with the relationships between the constructs (Chin, 2010; Hair Jr et al., 2014).

### 4.1 Evaluation of the measurement model

To evaluate the internal consistency of the measurement model, it was necessary to assess convergent validity and construct reliability. Convergent validity is acceptable if the loading of each indicator is >0.7 (Hair et al., 2011). A loading below 0.7 should be considered for item removal, provided that item removal allows increasing the composite reliability (CR) above 0.70 and also increasing the average variance extracted (AVE) above 0.5 (Chin, 2010; Hair Jr et al., 2014). Cronbach’s alpha coefficient was also considered for reliability assessment since CR and alpha values tend to be similar when using factor-based algorithms (Kock, 2015). Table 2 shows that all loadings of the 15 items of the present construct had a value above 0.90. Also, that the Alpha and CR values of all constructs had a value higher than 0.90 and, furthermore that all AVE values were higher than 0.80; therefore, the convergent validity of the measurement model was excellent.

**Table 2:** Assessment results of the measurement model.

Construto	Items	Loading	(α)	CR	AVE
Attitude (ATT)	ATT1	0.941	0.928	0.932	0.875
	ATT2	0.946			
	ATT3	0.919			
Subjective norms (SN)	SN1	0.870	0.877	0.879	0.804
	SN2	0.927			
	SN3	0.891			
Perceived behavioral control (PBC)	PBC1	0.939	0.942	0.943	0.895
	PBC2	0.954			
	PBC3	0.946			
Environmental awareness (EA)	EA1	0.723	0.867	0.876	0.875
	EA2	0.671			
	EA3	0.837			
	EA4	0.763			
	EA5	0.794			
	EA6	0.859			
Willingness to pay (WP)	WP1	0.951	0.945	0.945	0.900
	WP2	0.946			
	WP3	0.950			

Cronbach’s alpha (α) for all variables is >0.8, the composite reliability (CR) >0.70, the mean–variance extracted (AVE) >0.50, indicating a significant validity of the model.

To assess discriminant validity, Fornell-Larker criterion was used, thus, the square root of the AVE of each construct was calculated, which had to be greater than the highest correlation between the construct and other constructs in the model (Chin, 2010; Hair Jr et al., 2014). Table 3 shows that all values in the bold diagonal are greater than the correlations. Therefore, the measurement model meets all the assumptions necessary to continue with the evaluation of the structural model.

**Table 3:** Disciminant validity (Fornell-Lacker criterion).

	ATT	EA	PBC	SN	WP
Attitude (ATT)	**0.935**				
Environmental awareness (EA)	0.687	**0.777**			
Perceived behavior control (PBC)	0.777	0.637	**0.946**		
Subjective norms (SN)	0.720	0.557	0.770	**0.896**	
Willingness to pay (WP)	0.683	0.557	0.740	0.700	**0.949**

The square root of AVEs is shown diagonally in bold.

### 4.2 Evaluation of the structural model

Two criteria verified the evaluation of the structural model: (a) the significance of the path coefficients and (b) the value of the R2 coefficient for the endogenous constructs. To evaluate the structural model, the path coefficients for each relationship were calculated, as well as their corresponding *p*-values. The value of the R2 coefficient depends on the field of research (Chin, 1998) suggests values of 0.67, 0.33, and 0.19 as substantial, moderate, and weak measures of R, respectively. In behavior-related studies, a value of 0.2 for R2 is considered acceptable (Kock, 2013; Hair Jr et al., 2014). In the present work, the R2 coefficients for ATT, PBC and WP were 0.472, 0.406 and 0.0.603, respectively. That is, both R2 values were at high levels. Therefore, the values show that the variables in the present study explain a high percentage of the variance in WP.

**Figure 2:**
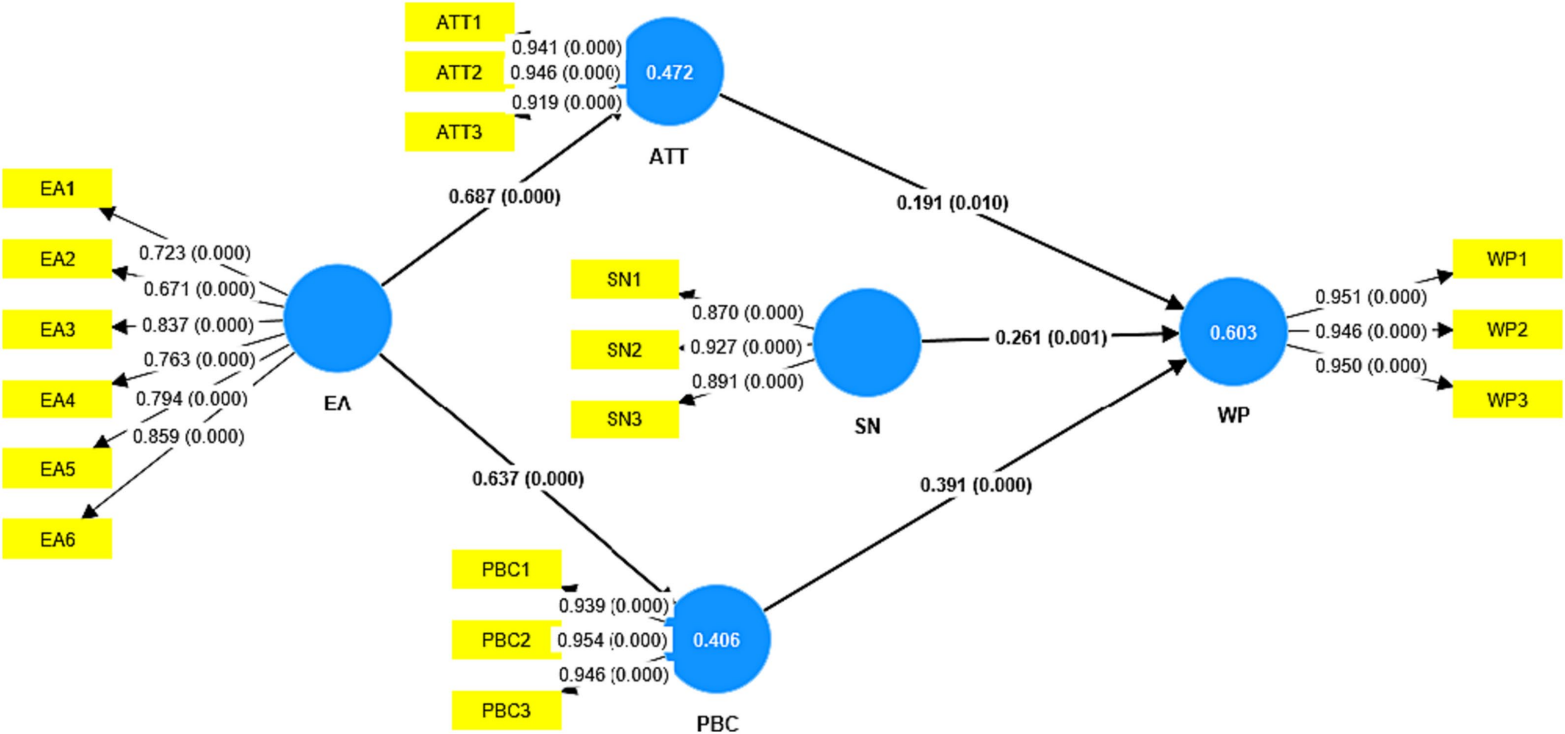
Structural model.

The hypothesis tests and the evaluation of the path coefficients can be seen in Table 4 and Figure 2. The results show the positive and significant effect of EA on ATT and PBC (*H1*, *H2*); also, the positive and significant effect of ATT, SN and PBC on WP (*H3*, *H4* and *H5*) was also demonstrated. In this way, all the hypotheses raised in the structural model were accepted.

**Table 4:** Hypothesis testing.

H	Hypothesis	Original	Sample	Standard	T statistics	*p* values	Decision
		sample (O)	mean (M)	deviation (STDEV)	(|O/STDEV|)		
*H1*	EA –> ATT	0.687	0.688	0.031	22.242	0.000	Supported
*H2*	EA –> PBC	0.638	0.639	0.033	19.208	0.000	Supported
*H3*	ATT –> WP	0.191	0.191	0.074	2.575	0.010	Supported
*H4*	PBC –> WP	0.391	0.390	0.069	5.703	0.000	Supported
*H5*	SN –> WP	0.260	0.263	0.079	3.291	0.001	Supported

## 5 Discussion

### 5.1 General discussion

In a broad sense, the findings of the study support the notion that environmental awareness (EA) has an influence on attitude (ATT) and perceived behavioral control (PBC). Also, the theory of planned behavior encompasses three key constructs: attitude (ATT), subjective norm (SN), and perceived behavioral control (PBC). Each of these constructs influences the willingness to pay (WP) for green products within the Peruvian market.

Specifically, the first hypothesis (*H1*) has been supported. The study shows that environmental awareness (EA) plays a fundamental role in influencing attitudes towards the consumption of green products. This finding suggests that more environmentally aware people are more likely to develop positive attitudes toward purchasing and using products with a lower environmental impact. This supports the notion that environmental awareness can be a crucial driver of change towards more sustainable consumption practices. The research supports Hypothesis 2, demonstrating that environmental awareness influences perceived behavioral control. This means that more environmentally conscious people feel more empowered to make decisions supporting sustainability in their purchasing choices. This relationship between environmental awareness and perceived control could significantly affect consumer habits and promote greater alignment between intentions and actions.

According to the results of hypotheses 1 and 2, these findings have important implications for marketing and promotion strategies for green products. By understanding how environmental awareness influences consumers’ attitudes and perceived behavior control, firms can adapt their approaches to encourage the adoption of sustainable products and promote environmental awareness among consumers. Furthermore, these results support the idea that environmental sustainability has become a relevant factor in consumer purchase decisions, highlighting the importance of offering green product options in the marketplace (Xu et al., 2020; Ali et al., 2021; Valenzuela-Fernández et al., 2022).

The findings of the study support the third hypothesis (*H3*), which postulates that attitudes (ATT) play a crucial role in influencing consumers’ willingness to pay (WP) for green products. Specifically, the results demonstrate that favorable attitudes towards these items have a statistically significant and beneficial impact on customers’ desire to pay for them. Hence, when individuals possess favorable attitudes toward environmentally friendly products, they are more inclined to pay a higher price. This highlights the significance of efforts to enhance consumer perceptions about environmental sustainability to augment the inclination to support eco-friendly items financially.

The findings mentioned in *Hypothesis 3* line up with Gomes et al. (2023) study, which looks at Generation Z’s attitudes toward the higher price tags they are willing to pay for environmentally friendly products. The consumer places a lot of importance on an item’s environmental attributes and is willing to pay more due to the widespread use of digital technology, increased environmental awareness, and a strong emphasis on sustainability.

The fourth hypothesis (*H4*) focused on investigating the potential impact of subjective norms (SN) on customer willingness to pay for green products (WP). The test findings suggest a statistically significant and positive correlation between personal norms and individuals’ willingness to pay. This finding implies that customers’ social perceptions and expectations within their immediate surroundings impact their inclination to pay more for environmentally friendly items. In essence, when individuals perceive that their societal context places importance on and provides backing for acquiring environmentally sustainable products, they demonstrate a greater inclination to pay extra costs to get these products.

The results of *Hypothesis 4* match prior research, which affirms that there is a positive correlation between social factors and the preference towards adopting environmentally responsible behavior (García-Salirrosas et al., 2023; Valenzuela-Fernández et al., 2023). This finding provides an opportunity to evaluate the influence of personal norms on the willingness to pay for green products.

Finally, the fifth hypothesis (*H5*) was examined to determine the potential impact of perceived behavioral control (PBC) on customers’ willingness to pay for green products (WP). The research results show a statistically significant link between perceived behavioral control (PBC) and willingness to pay (WP), which supports the hypothesis. This implies that the degree to which consumers believe they have control over their capacity to make purchasing choices for environmentally friendly items impacts their willingness to pay a higher price for such products. The perception of being well-informed and able to influence their sustainable purchasing decisions is a driving force behind their willingness to pay a higher fee for environmentally friendly items.

In addition, these results of Hipotesis 5 align with earlier research that shows how important perceived behavioral control (PBC) is for consumers to form long-lasting buying habits, as shown in the study by Shi and Jiang (2023). Additionally, research by Peluso et al. (2021) and Alexa et al. (2021) supports the growing trend toward the adoption of sustainable and environmentally friendly products.

Finally, the findings are congruent with sustainable practices within the Latin American context. There has been an observed change in consumer perception, resulting in a corresponding rise in demand (Madrigal-Moreno et al., 2021; Severo et al., 2021; Valenzuela-Fernández et al., 2022). Hence, an extensive understanding of customers’ buying intentions could help local manufacturers formulate efficacious tactics to enhance their competitiveness within the market (González-Cabrera and Trelles-Arteaga, 2021; García-Salirrosas and Rondon-Eusebio, 2022; Gómez-Bayona et al., 2023). The interactions between consumers and brands are important in forecasting product and service purchases and purchase intentions. Therefore, effective information management significantly influences consumer decision-making and the consumption patterns of certain items (Lopes et al., 2023).

### 5.2 Implications

In the post-pandemic society, consumers are increasingly aware of environmental issues and are more willing to pay for products perceived as sustainable when environmental awareness is actively promoted (Moorthy et al., 2023). However, purchasing decisions remain uncertain (Kim and Lee, 2023). Therefore, the results obtained in this study may have implications in the field of marketing and brand positioning, as consumers can make more informed purchasing decisions by aligning their environmental values with their consumption choices as evidenced in the literature.

Companies can take advantage of this knowledge by developing marketing strategies that highlight the sustainable aspects of their products and align with the values of environmentally conscious consumers. Peruvian companies can use these results to design specific information campaigns and promote sustainable practices in their markets, which would improve local producers’ competitiveness. In particular, the results contribute to Peruvian industries and can be replicated and adapted to other geographical contexts.

Furthermore, businesses and public institutions must promote environmental education and awareness. Because environmental awareness can directly impact shaping a more sustainable society in its consumer choices, consumers aware of the effects of green consumption are more willing to pay for them. From a policy and regulatory perspective, this study supports the need to implement policies that promote sustainability and environmental responsibility. Promoting the creation and use of green products could cause a big change in how people consume products in a way that is better for the environment. This supports the idea that people need to be more aware of the environment for responsible and long-lasting consumption habits to spread, which is good for everyone and the environment. The results contribute to the discourse on building a more environmentally conscious and responsible society, thus promoting a sustainable consumption model. Likewise, policymakers and environmental organizations can use these results to promote awareness of sustainable consumption patterns.

## 6 Conclusión

This study has effectively shown the significant impact of environmental awareness on consumer behavior and its influence on willingness to pay for green products in the Peruvian market. Based on the theory of planned behavior, the study shows that being aware of the environment positively affects your attitudes and perceived behavior control. The research results show a statistically significant link between these critical factors and environmental awareness. This shows how consumers can be more willing to pay for eco-friendly products. For this reason, environmental awareness improves transformative consumer practices, fostering sustainability and ecological responsibility. Because of this, a consumer who cares about the environment is likely to carefully think about how a product is made, how it is distributed, what it is made of, and how it affects sustainability, reusability, and climate change.

This model provides empirical evidence for the impact of environmental awareness on consumer behavior, specifically in purchasing decisions and willingness to pay for environmentally friendly items. Notably, environmentally responsible behavior emerges as a pivotal element shaping consumer willingness to pay for green products, aligning with the findings of Kaiser et al. (1999) and Omarova and Jo (2022). Recent events, like COVID-19, have made this trend stronger. As noted in the literature review, previous studies have shown that people want more organic and health-promoting products with eco-friendly features and high-quality materials.

This underscores the evolving consumer landscape, where environmental considerations are increasingly integral in shaping purchasing decisions, reflecting a growing consciousness towards sustainability and health in the Peruvian market. Furthermore, the aftermath of COVID-19 has amplified these effects, with health and well-being concerns further motivating consumers to favor environmentally friendly options, even at higher prices. Social norms also come into play, as positive attitudes towards sustainability correlate with an increased willingness to pay for eco-friendly products.

### 6.1 Limitations and future research

This research should consider some limitations for future research. The first refers to the type of sampling, which is non-probabilistic and transversal and includes only consumers from Peru, which could limit the conclusions obtained and the generalizability of the study results. Secondly, the data collection method was conditioned by the limitations of COVID-19. The study was conducted through an online survey in which participants responded voluntarily, which did not allow the evaluation of consumer behavior in real-time. Third, the temporality of the study, by not allowing the results to be extrapolated to post-pandemic scenarios, makes it possible to explore opportunities for future studies that evaluate the evolution of COVID-19 in post-pandemic scenarios. A final limitation that suggests observing the generalization of the study’s conclusions is related to the honesty in the responses of individuals, a product of social pressure and ethics related to issues of environmental interest.

This study makes it possible to provide preliminary information on consumer behavior for future studies of gaps between purchase intention and purchase experience, considering that many of the consumers who declare their intention to purchase sustainable products commonly end up not buying them (Severo et al., 2021; Valenzuela-Fernández et al., 2022; García-Salirrosas et al., 2023; Valenzuela-Fernández et al., 2023).

## Data availability statement

The original contributions presented in the study are included in the article/supplementary material, further inquiries can be directed to the corresponding authors.

## Author contributions

EEG-S: Conceptualization, Data curation, Formal analysis, Funding acquisition, Investigation, Methodology, Project administration, Resources, Software, Supervision, Validation, Visualization, Writing – original draft, Writing – review & editing. ME-F: Conceptualization, Investigation, Resources, Validation, Writing – original draft, Writing – review & editing. LG-B: Investigation, Resources, Supervision, Validation, Visualization, Writing – original draft. GM-L: Conceptualization, Investigation, Project administration, Resources, Writing – review & editing. AV-A: Conceptualization, Project administration, Resources, Supervision, Visualization, Writing – original draft, Writing – review & editing. RG-C: Conceptualization, Funding acquisition, Project administration, Resources, Supervision, Validation, Visualization, Writing – review & editing.
